# Genome Sequences of Two Single-Stranded DNA Viruses Identified in *Varroa destructor*

**DOI:** 10.1128/genomeA.00107-18

**Published:** 2018-03-01

**Authors:** Simona Kraberger, Gabriel A. Visnovsky, Ron F. van Toor, Maketalena F. Male, Kara Waits, Rafaela S. Fontenele, Arvind Varsani

**Affiliations:** a The Biodesign Center for Fundamental and Applied Microbiomics, Center for Evolution and Medicine, School of Life Sciences, Arizona State University, Tempe, Arizona, USA; b Department of Chemical and Process Engineering, Biomolecular Interaction Centre, University of Canterbury, Christchurch, New Zealand; c The New Zealand Institute for Plant and Food Research Limited, Christchurch, New Zealand; d School of Biological Sciences, Biomolecular Interaction Centre, University of Canterbury, Christchurch, New Zealand; e School of Environmental and Life Sciences, The University of Newcastle, Callaghan, New South Wales, Australia; f Structural Biology Research Unit, Department of Clinical Laboratory Sciences, University of Cape Town, Cape Town, South Africa

## Abstract

Varroa destructor is a ubiquitous and parasitic mite of honey bees, infecting them with pathogenic viruses having a major impact on apiculture. We identified two novel circular replication-associated protein (Rep)-encoding single-stranded (CRESS) DNA viruses from V. destructor sampled from a honey bee hive near Christchurch in New Zealand.

## GENOME ANNOUNCEMENT

Varroa destructor is a parasitic mite that feeds on the hemolymph of honey bee (Apis mellifera). This mite is implicated in colony collapse and transmits several viruses (1). So far, only positive-sense RNA viruses belonging to the Dicistroviridae and Iflaviridae genera (order Picornavirales) have been associated with V. destructor, and these viruses also infect honey bees (2, 3). However, DNA viruses associated with V. destructor have yet been identified. To determine if DNA viruses are present, 10 V. destructor individuals were collected from a honey bee (A. mellifera ligustica) hive near Christchurch (New Zealand; 43.6401°S, 172.4842°E) on 1 July 2015, 15 years after Varroa was first identified in New Zealand (4).

The Varroa specimens were pooled and homogenized with 1 ml of SM buffer (0.1 M NaCl, 50 mM Tris-HCl [pH 7.4]). The homogenate was centrifuged at 10,000 × *g* for 5 min and the supernatant filtered through a 0.2-µm-pore-size syringe filter. Viral DNA was extracted from the filtrate using a High Pure viral nucleic acid kit (Roche Diagnostics, USA), followed by amplification of circular DNA molecules using the illustra TempliPhi kit (GE Healthcare, USA) and sequencing in a Illumina HiSeq 2500 platform at Novogene, Hong Kong. The paired-end reads were *de novo* assembled using ABySS 1.9 (5), and contigs >500 nucleotides (nt) were analyzed against a viral sequence database. Two contigs sharing similarities to circular replication-associated protein (Rep)-encoding single-stranded (CRESS) DNA viruses were identified. Abutting primers were designed and used together with a HiFi HotStart DNA polymerase (Kapa Biosystems, USA) to amplify full viral genomes. These were cloned and Sanger sequenced at Macrogen, South Korea.

CRESS DNA viruses are classified into eight recognized families, and a large number are currently unclassified. Various CRESS DNA viruses have been identified in various insect samples, including whiteflies (6, 7), mosquitoes (8), dragonflies and damselflies (9–11), ticks (12), and thrips (13). Of the two CRESS DNA viruses identified, one is a member of a recently established virus family, Genomoviridae (14, 15), and the other is a novel virus. Varroa mite associated genomovirus 1 (VmaGV-1; 2,194 nt), with a TAATATTAT nonanucleotide motif, shares 77.3% genome-wide pairwise identity with Pacific flying fox feces-associated gemycircularvirus-9 (PfffaGmV-9; GenBank accession no. KT732803) and thus is a member of the genus Gemycircularvirus of the family Genomoviridae. VmaGV-1 encodes three proteins, a capsid protein (CP) in the virion sense and a Rep and RepA in the complementary sense that share 46%, 84%, and 94% amino acid identity with the CP, Rep, and RepA of PfffaGmV-9, respectively. The second genome, Varroa mite associated virus 1 (VmaV-1; 1,811 nt), with a CAGTATTAC nonanucleotide motif, is a novel CRESS DNA virus that encodes a Rep and CP in the virion sense. This genome shares low similarity to other known viruses and is most closely related to Giant panda circovirus 4 (GenBank accession no. MF327576), sharing 60% genome-wide identity, 36% amino acid identity to the Rep, and no similarities to any known CP. The two genome sequences described here represent the first documented DNA viruses to be associated with V. destructor, and the pathogenicity of these viruses in Varroa or their parasitized host, honey bees, is currently unknown.

### Accession number(s).

The complete genome sequences of Varroa mite-associated genomovirus 1 isolate VPVL_36 and Varroa mite-associated virus 1 isolate VPVL_46 were deposited in GenBank with accession numbers MG571087 and MG571088, respectively.
